# Association of Placental Histology with the Pulsatility Index of Fetal and Uteroplacental Vessels during Pregnancy and with Birthweight Z-Score

**DOI:** 10.18103/mra.v11i8.4238

**Published:** 2023-08-31

**Authors:** Hein Odendaal, Lut Geerts, Colleen Wright, Drucilla J Roberts, Pawel Schubert, Theonia K Boyd, Lucy Brink, Daan Nel

**Affiliations:** a Department of Obstetrics and Gynaecology, Stellenbosch University, Cape Town, South Africa; b Lancet Laboratories, Johannesburg, South Africa; c Division of Anatomical Pathology, Tygerberg Hospital, National Health Laboratory Service, Faculty of Medicine and Health Sciences, Stellenbosch University, Cape Town, South Africa; d Department of Pathology, Massachusetts General Hospital, Boston, Massachusetts, USA; e Department of Pathology, Division of Anatomic Pathology, Texas Children’s Hospital, Houston, Texas, USA; f Department of Statistics and Actuarial Science, Stellenbosch University, Stellenbosch, South Africa

**Keywords:** Placental histology, multivessel Doppler assessment, maternal vascular malperfusion, fetal vascular malperfusion, placental abruption, birthweight z-score

## Abstract

**Aims:**

To compare macro- and microscopic features of the placenta with the pulsatility index (PI) of the uterine (UtA), umbilical (UA) and middle cerebral arteries at 20-24- and 34-38-weeks’ gestation, and with birthweight z-scores (BWZS).

**Methods:**

Recruitment for the Safe Passage Study, which investigated the association of alcohol and tobacco use with stillbirth and sudden infant death syndrome, occurred from August 2007 to January 2015 at community clinics in Cape Town, South Africa. The population represents a predominantly homogenous population of pregnant women from a low socioeconomic residential area. This study is a further analysis of the data of the Safe Passage Study. It consists of 1205 singleton pregnancies for which placental histology was available, of whom 1035 had a known BWZS and 1022 and 979 had fetoplacental Doppler examinations performed at Tygerberg Academic Hospital at 20-24 and 34-38 weeks respectively. Features of the placenta were assessed according to international norms.

**Results:**

Significantly higher ORs for the presence of individual and combined features of maternal vascular malperfusion (MVM) were found with lower BWZS and higher UtA PI values, more consistently than with higher UA PI values. Strongest associations were for a small placenta for gestational age (UtA OR 4.86 at 20-24 and 5.92 at 34-38 weeks; UA OR 5.33 at 20-24 and 27.01 at 34-38 weeks; low BWZS OR 0.31), for accelerated maturation (UtA OR 11.68 at 20-24 weeks and 18.46 at 34-38 weeks; low BWZS 0.61), for macroscopic infarction (UtA OR 6.08 at 20-24 weeks; UA OR 17.02 at 34-38 weeks; low BWZS OR 0.62) and for microscopic infarction (UtA OR 6.84 at 20-24 and 10.9 at 34-38 weeks; low BWZS OR 0.62).

**Conclusion:**

There is considerable variability in the associations between individual features of MVM and increased UtA or UA PI and low BWZS. Although all MVM features currently carry equal weight in defining the condition of MVM, our data suggest that some should carry more weight than others. Macroscopic examination of the placenta may be helpful in identifying placental insufficiency as a small placenta for gestational age and macroscopic infarction were the features most strongly associated with outcomes.

## Introduction

Although placental insufficiency affects about 3.17% of pregnancies, it has until recently not been studied adequately.^1^ According to a recent meta-analysis of 29 studies, placental pathology was responsible for 11% to 65% of stillbirths,^2^ but only three of these studies were conducted in low-income countries where most stillbirths occur.^3^ Redline emphasizes that it is essential for clinicians to appreciate the pivotal role placental pathology plays in understanding adverse pregnancy outcome.^4^ As adequate development of the placental villous tree is essential for fetal growth, it is important to establish how this process is affected by environmental conditions,^5^ especially since maternal vascular malperfusion (MVM) of the placenta has been reported as the most common cause of stillbirth.^6,7^

The histologic lesions of MVM represent both primary vascular pathology due to abnormal spiral artery remodeling, and secondary villous pathology as a response to a combination of abnormal oxygen tension and oxidative stress.^8^ Paules et al. found that fetoplacental Doppler parameters, measured by the pulsatility index (PI) within two weeks of delivery, were associated with maternal-side vascular lesions of the placenta, mainly malperfusion.^9^ Although the features of secondary villous pathology such as villous infarction, accelerated villous maturation and distal villous hypoplasia were referred to, it is still uncertain how individual pathological features relate to Doppler flow velocity waveforms of the uterine (UtA), umbilical (UA) and mid-cerebral arteries (MCA) at different gestations.

It is necessary to understand the placental pathology associated with abnormal Doppler waveforms better: (i) as UtA PI is negatively associated with remodeling of the spiral arteries or smaller vessels branching from the UtA;^10^ (ii) as UA PI is frequently used to distinguish between the growth-restricted and constitutionally small fetus;^11^ and (iii) as abnormal MCA PI is associated with adverse cognitive outcome in very-low-birthweight infants.^12^ It is also important to note that there is still uncertainty about the best combination of findings to represent each injury pattern of the placenta.^4,13^ Information from the Safe Passage Study (SPS), a prospective longitudinal study to investigate the effects of smoking and drinking during pregnancy on stillbirths and sudden infant deaths,^14^ provided us with a unique opportunity to study the placenta in normal and abnormal pregnancies.^15^ As placental abruption, MVM, fetal vascular malperfusion (FVM), and infection were the most common causes of stillbirths in SPS (25.5%, 16.6%, 15.9%, and 13.8% respectively),^16^ we decided to investigate the association of fetal growth and the PI of the UtA, UA, and MCA at different time points, with macroscopic and microscopic placental features at delivery.

## Methods

**Figure 1.**
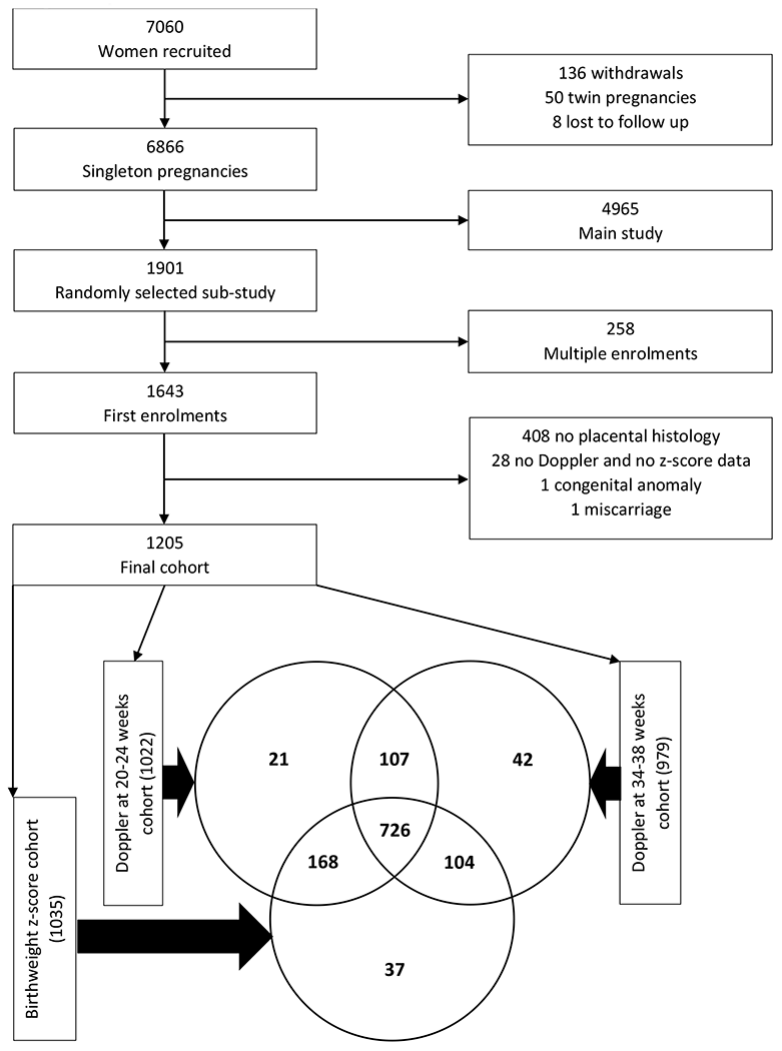
Study profile

Pregnant women were recruited for the SPS in the USA and in South Africa (SA).^14^ The South African cohort included women of low socioeconomic status who attended antenatal clinics in a geographically well-defined residential area close to Tygerberg Academic Hospital between August 2007 and January 2015 (N=7 060). The study was explained to women waiting for their first antenatal visit and the first five women of the day willing to participate were recruited. Participants who were less than 24 weeks pregnant at enrolment, were eligible for random selection by a computer-generated number (28% of enrolled participants (N = 1 916) for the SA cohort) to participate in additional prospective investigations, including ultrasound examinations at 20-24 and 34-38 weeks for fetal biometry and Doppler PI of the UtA, UA, and MCA, and collection of the placenta for macro- and microscopic examination. For this secondary analysis, only the SA data were used to avoid combining different populations. Only women with singleton pregnancies AND histological examination of the placenta were included and if women were enrolled in the study for two or more pregnancies, only the first one was included in this analysis as multiple enrolments could introduce bias.^17^ Not all of the 1 205 eligible participants attended all visits. (Figure 1).

Findings from the earliest ultrasound examination, before or at recruitment, were used to calculate the gestational age (GA) as this has been demonstrated to be the most accurate dating method in this community.^18^ Cases without BWZS (if GA at delivery was before 168 or after 299 days or if infant birthweight or sex was not recorded) and those lacking a dating scan before 20 weeks were excluded from the BWZS cohort (N = 170). During the preliminary feasibility study an ultrasound manual was developed with detailed instructions for the different Doppler and biometric measurements. Quality of the measurements was assessed on initial saved images of all three sonologists involved in the study.

### For all Doppler measurements,

the wall filter was set at less than 100 Hz and baseline and pulse repetition frequency (PRF) were adjusted to eliminate aliasing and display maximal trace size, filling at least 50% of the image in single screen display. The arterial PI was measured by automated electronic tracing on at least three consecutive similar waveforms of crisp quality obtained with a minimal (less than 30 degrees) angle of insonation. For UtA assessment, both left and right UtA were interrogated with a 5 mm sample volume placed within 1 cm and anterior to the apparent crossover of the UtA and external iliac artery seen with colour mode. Presence or absence of early diastolic notching was noted for each vessel and the mean (of left and right) UtA PI was used for this analysis. For UA assessment, a free loop of umbilical cord was identified, halfway between the fetal and placental ends. The most vertical portion of the vessel was sampled with a 5 mm sample volume, placed in the lumen of one UA, including a portion of the vein which had to have a ‘monophasic’ flow pattern to confirm fetal quiescence. The presence (forward or reversed) or absence of the end diastolic flow (EDF) was also recorded. For MCA assessment, the vessel was visualized in an enlarged transverse axial view of the fetal head, slightly more caudal than the biparietal diameter section without undue pressure on the head. Colour Doppler was used to identify the MCA of the proximal hemisphere as a major lateral branch of the circle of Willis coursing anterolaterally, adjacent to the lesser wing of the sphenoid bone, at the border between the anterior and middle cerebral fossa, and towards the transducer. A 5 mm gate was placed over the lumen of the MCA close to the circle of Willis, within the proximal half of the MCA.^19^

### Placentas

were placed in 10% buffered formalin and examined macroscopically at the pathology laboratory according to standardized protocols as published previously^20,21^ and listed in Appendix A. Placental tissues were sampled and routinely processed to hematoxylin and eosin-stained slides for examination. Two experienced perinatal pathologists (CW, PS) examined the slides and reported according to a standard template (Appendix B). To prevent bias the only clinical information that was provided was the GA at delivery and whether the infant was born alive or not. For stillbirths, the slides were also examined by two external perinatal pathologists (TB, DR). The conclusion was a summation of the macroscopic and microscopic features found, and this was subsequently adapted to comply with the Amsterdam Placental Workshop Group Consensus Statement, when this became widely available in 2016.^22^ Prior to and during the study quality control was ensured by checking interobserver concordance between all pathologists, on placentas randomly selected by the monitoring site, with a standard of 90% agreement for individual items. Diagnosis of MVM was based on a constellation of macroscopic and microscopic findings, 12 in total, not all of which were present in every individual case. A minimum of three items were required to allocate a diagnosis of MVM. Macroscopic findings of MVM included a small placenta for GA (trimmed placental weight < P10 for gestation^23^), macroscopic infarction and retroplacental hemorrhage (RPH), the pathological term corresponding to a clinical diagnosis of placental abruption. Macroscopic infarction was defined as firm solid areas extending from the basal plate with the apex towards the fetal surface. Any infarction in a preterm placenta and > 5% of the placental surface at term, were regarded as pathological. Macroscopic RPH was defined as an indentation of > 15% on the maternal surface of the placenta, often with gross intervillous hemorrhage. Microscopic placental features of MVM included microscopic RPH or infarction, distal villous hypoplasia, accelerated villous maturation, decidual arteriopathy and increased syncytial knots. Microscopic RPH was defined by the presence of blood beneath the decidua and dissecting into the decidua and placental parenchyma, with congestion and/or intravillous hemorrhage, sometimes with additional coagulation necrosis of the syncytiotrophoblast nuclei with overlying infarction. Criteria for distal villous hypoplasia included small and stringy villi, elongated, or tiny, barely the size of a syncytial knot. This could be focal or diffuse and was usually only seen in placentas < 32 weeks. Accelerated villous maturation was diagnosed when villi were hypermature for a known GA and usually associated with an increase in syncytial knots (size and number). Decidual arteriopathy refers to an absence of spiral arterial remodelling with mural hypertrophy only in the basal plate vessels or with fibrinoid necrosis with foam cells in the decidua capsularis/parietalis. Increased syncytial knots was defined as their presence in > 33% of villi at term. Increased perivillous fibrin and increased extravillous trophoblast (IEVT), previously regarded as features of MVM, are now no longer part of the Amsterdam consensus criteria for MVM but were still recorded in this study.^22^

Features of FVM included avascular villi, chorionic plate/stem villous thrombosis, microscopic thrombosis of the umbilical cord, and villous-stromal karyorrhexis. A minimum of two criteria were required for the diagnosis of FVM.^22^

Features of inflammation included chorioamnionitis (ascending infection), and villitis which included infectious and non-infectious villitis (villitis of unknown etiology (VUE)). Chorioamnionitis was staged and graded per Redline^4^ and additional features such as subchorionic micro-abscesses were noted. Villitis was recorded as acute (neutrophilic), or chronic (lymphocytic/histiocytic). If chronic features, such as plasma cells or an accompanying funisitis or viral inclusions, were present that might suggest a specific or infective etiology, further investigations were undertaken. The remainder were recorded as VUE and graded as focal, multifocal or diffuse, and the presence of obliterative fetal vasculopathy was noted as it has prognostic significance.^24,25^

Fetal growth was assessed by the BWZS for GA at delivery (168-299 days) using sex-adjusted standards of the INTERGROWTH–21^st^ study, as these standards are similar for different countries and continents.^26^

The odds ratios (ORs) of abnormal placental features with PI values at 20-24 and 34-38 weeks’ gestation of the UtA, UA and MCA, and with the BWZS, were then determined.

### Statistical analyses.

Pulsatility index values of the UtA, UA and MCA at 20-24 weeks’ and 34-38 weeks’ GA and the BWZS were associated with the individual and overall placental features using STATISTICA (Dell Inc. (2015) Dell Statistica (data analysis software system), version 13. software.dell.com). Continuous variables were compared to nominal variables with analysis of variance (ANOVA). For dichotomous nominal variables the relationship with continuous variables were also investigated with logistic regression where odds ratios were computed and reported with 95% confidence intervals. Pearson correlations were used for the comparison of continuous variables.

## Results

The study consists of 1 205 women with gross and histological examination of their placentas, of whom 1 022 and 979 had Doppler examinations at 20-24 and 34-38 weeks respectively, and 1 035 had a calculable BWZS (Figure 1). Some participants lacked one or both Doppler examinations, or BWZS, resulting in three different but overlapping study groups. The prevalence of pregnancy-induced hypertension, preeclampsia, and gestational diabetes was 4%, 4%, and 1% respectively. Sixty-six percent of women reported smoking cigarettes, 68% the use of alcohol and 11% the use of recreational drugs. The prevalence rates of low birthweight (< 2500 g), small for GA infants (< 10^th^ percentile) and preterm birth (before 37 weeks) were 15%, 17%, and 13% respectively. Demographic maternal data of the three study groups are listed in Table 1. The group with Doppler results at 34-38 weeks’ gestation included only 3 of the 15 stillbirths and no deliveries < 34 weeks and had a higher birthweight, GA at delivery and BWZS.

The most frequent placental findings were a small placenta for GA, increased syncytial knots, increased perivillous fibrin, MVM, chorioamnionitis, and villous-stromal karyorrhexis. Placentas with Doppler results at 34-38 weeks group had a lower rate of accelerated maturation and higher rate of increased syncytial knots compared to the other two groups, and less villitis (98.3% due to VUE) than those with Doppler results at 20-24 weeks. Cases with Doppler assessment at 20-24 weeks group had a higher prevalence of a small placenta for GA and chorioamnionitis than the BWZS group (Table 2).

The ORs associated with UtA PI at 20-24 weeks, were significantly higher for all currently accepted individual features of MVM and IEVT as well as the composite MVM diagnosis (based on three or more features), villous-stromal karyorrhexis, and villitis (Table 3). At 34-38 weeks the ORs were significantly higher for small placenta, microscopic pathological infarction, accelerated maturation, distal villous hypoplasia, increased syncytial knots, MVM and villitis (with a trend for IEVT).

The ORs associated with UA PI at 20-24 weeks were significantly higher for a small placenta for GA, MVM and villous-stromal karyorrhexis, increased syncytial knots and IEVT (Table 4). At 34-38 weeks, the ORs were significantly higher for a small placenta, macroscopic pathological infarction, decidual arteriopathy, distal villous hypoplasia and MVM. (Table 4).

At 20-24 weeks the only significant finding in the MCA was a higher OR with macroscopic RPH (Table 5). At 34-38 weeks, the OR was significantly lower for a small placenta and any retroplacental haemorrhage (trends for macroscopic and microscopic RPH), with trends for a higher OR for increased syncytial knots, and distal villous hypoplasia.

The ORs associated with BWZSs were significantly lower for all the macro- and microscopic features of MVM and for villous-stromal karyorrhexis, all with narrow confidence intervals. The most significant reduction was in the prevalence of a small placenta for gestational age, followed by accelerated maturation, macroscopic and microscopic pathological infarction (Table 6).

**Figure 2.**
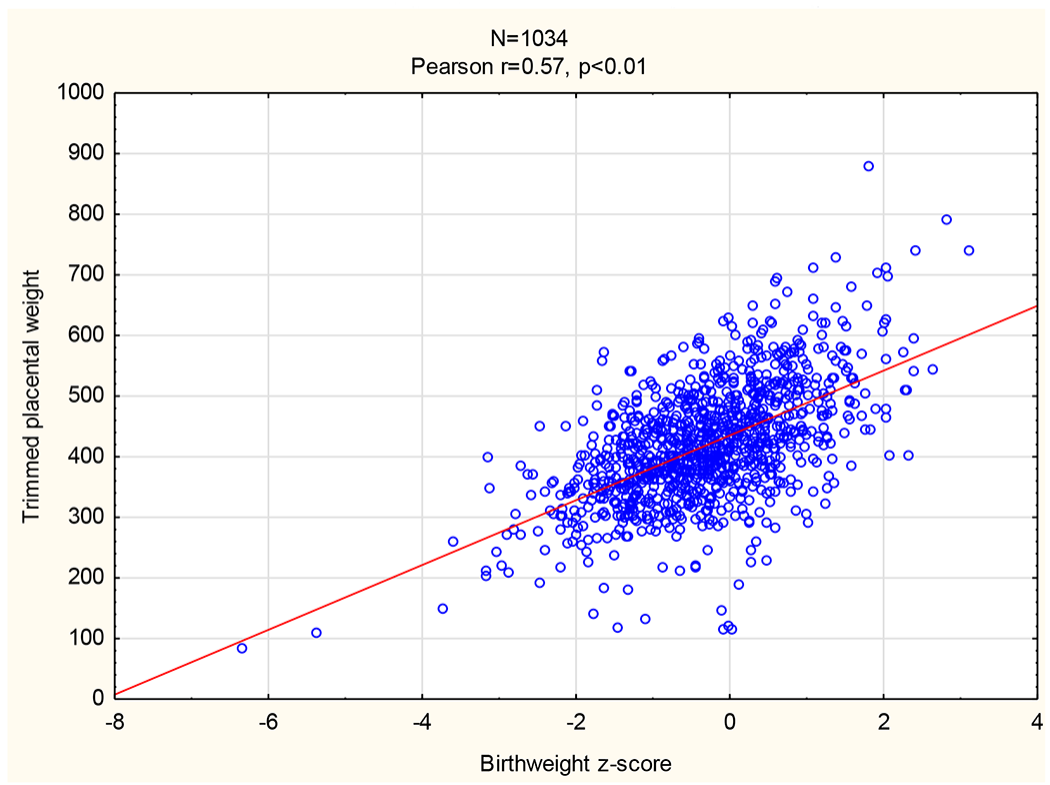
Correlation between birthweight z-score and trimmed placental weight.

Pearson correlations were used to compare trimmed placental weight with PI values of the UtA, UA, and MCA and with BWZS. At 20-24 weeks the UtA (Pearson *r* = −0.29, p < 0.01) and UA PI (*r* = −0.20 (p < 0.01) correlated negatively with trimmed placental weight but MCA PI was not correlated (*r* = −0.03). At 34-38 weeks’ gestation all three arterial PIs correlated significantly with trimmed placental weight (*r* = −0.19 (p < 0.01), −0.29 (p < 0.01) and 0.12 (p < 0.01), respectively). Birthweight z-scores had the strongest correlation with the trimmed placental weight (Pearson r=0.57; p < 0.01) (Figure 2).

## Discussion

At 20-24 weeks, increasing UtA PI was associated with significantly higher ORs for all currently accepted features of MVM: accelerated maturation (OR 11.68), microscopic (OR 6.84) and macroscopic (OR 6.08) infarction, a small placenta for GA (OR 4.86), decidual arteriopathy (OR 2.23), distal villous hypoplasia (OR 1.82) and increased syncytial knots (OR 1.75)), as well as the composite diagnosis of MVM (OR 3.23), IEVT (OR 1.63), villous-stromal karyorrhexis (OR 1.88) and villitis (OR 1.85). At 34-38 weeks significantly increased ORs applied to a smaller number of MVM features: accelerated maturation (OR 18.46), microscopic infarction (OR 10.9), a small placenta for GA (OR 5.92), increased syncytial knots (OR 3.28), distal villous hypoplasia (OR 3.12), as well as the composite diagnosis of MVM (OR 2.67) and villitis (OR 3.30). Compared with the UtA PI, increasing UA PI demonstrated fewer significant differences regarding placental findings; at 20-24 weeks only for a small placenta for GA (OR 5.33), villous-stromal karyorrhexis (OR 2.62), and MVM (OR 2.53); and at 34-38 weeks for a small placenta for GA (OR 27.01), macroscopic infarction (OR 17.02), decidual arteriopathy (OR 5.97), distal villous hypoplasia (OR 4.97), and MVM (OR 2.62). For the MCA few significant differences were found, at 20-24 weeks a higher prevalence of macroscopic RPH (OR 2.6) and at 34-38 weeks a lower incidence of any RPH (OR 0.53) and a small placenta for GA (OR 0.54). All the features of MVM and villous stromal karyorrhexis correlated significantly with lower BWZS.

Since most placental disease processes are not confined to or defined by a single pathological feature,^27^ it is essential to determine which lesions are most strongly associated with commonly used assessments of placental development and function such as Doppler velocimetry, and with outcomes such as growth restriction, as we have done in this study. We were able to rank placental findings according to their effects on BWZS and found the strongest correlations between BW deficit and a small-for-gestation placenta, accelerated maturation, macro- and microscopic placental infarction, and a composite diagnosis of MVM. Features of MVM correlated significantly with BWZS, in contrast to the other groups of placental lesions, thereby confirming that MVM is the main cause of fetal growth restriction.

The finding that the PI of the UtA at 20-24 weeks was significantly higher for all currently accepted features of MVM supports the observation that UtA waveform analysis in the second trimester can identify women at risk for severe adverse outcomes.^28^ Uterine artery PI was highest for accelerated maturation and macroscopic and microscopic infarction, followed by a small placenta.

The fewer significant associations of a higher UtA PI at 34-38 weeks compared with 20-24 weeks could be due to more advanced maternal cardiovascular remodelling occurring later in pregnancy.^13,29^ Other explanations could be that women with complicated pregnancies were more often delivered before the scheduled Doppler examination at 34-38 weeks (leading to a narrower distribution in PI values) or that defective remodelling of the spiral arteries is more common in early compared to late fetal growth restriction.^30^

The contrary was found for UA PI since it was only significantly higher for a small-for-GA, trimmed placental weight, and MVM, but not for the other individual features of MVM at 20-24 weeks, yet at 34-38 weeks UA PI values were significantly higher for several additional MVM features including macroscopic infarction, decidual arteriopathy and distal villous hypoplasia. This occurred despite all deliveries before 34 weeks and 80% of all stillbirths (likely with the worst placental pathology) being excluded from this assessment, which would have weakened the association. This illustrates the increase in placental lesions with advancing GA and the UA PI being sensitive to the secondary changes resulting from earlier hypoxia.

A decrease in the MCA PI in the context of placental insufficiency occurs due to vasodilatation in response to progressive fetal hypoxemia. This is a compensatory mechanism to ensure preserved oxygenation to essential organs like the brain, and it usually precedes delivery only by a few weeks when placental adaptation has failed to maintain a normal partial pressure of oxygen in the fetal circulation. As such, MCA PI changes occur at a more advanced stage of the underlying placental disease than either UtA or UA PI, and changes typically occur after 24 weeks.^31,32^ Our findings are in keeping with this as at 20-24 weeks (remote from delivery), a lower MCA PI was not found with any of the placental lesions while at 34-38 weeks PI values were lower with a small placenta and any RPH.

Of all MVM features, only a small-for-GA placenta was associated with worse Doppler results for all three vessels (MCA only later) and with lower BWZS, indicating that it can be a useful surrogate marker for fetal growth restriction. The significant correlation of a small placenta for GA with arterial PI values and with BWZS is in line with a previous finding that a small placenta is associated with higher uterine resistance to blood flow in the first trimester,^33^ and it justifies this feature being included as a diagnostic marker for MVM. Local growth charts for placental weight were not available and more than half of the placentas were considered small for GA using data from other populations.^23^ As small placentas were associated with higher PI values and with lower BWZS, this observation may not be due to ethnic differences between populations but possibly related to harmful exposures like cigarettes and alcohol, which are common in this community,^34^ and therefore a possible surrogate marker for fetal risk.

Our findings are in line with a state-of- the-art review indicating that Doppler evaluation of the placenta greatly contributes to the diagnosis and management of various placental lesions.^35^ Accelerated maturation had one of the strongest associations with higher UtA PI at both visits and was the individual characteristic with the second strongest association with BWZS, after a small-for-gestation placenta. The underlying pathogenesis for accelerated maturation is defective remodelling of the spiral arteries causing malperfusion of the placenta with subsequent decreased surface area and damage to the syncitiotrophoblast.^36^ Accelerated villous maturation is commonly associated with increased syncytial knots and distal villous hypoplasia, two other independent MVM lesions.^37^ Accelerated villous maturation occurs more frequently in spontaneous preterm labour (13.2%) when compared to uncomplicated pregnancy (0%)(adjusted p<0.001).^38^ In another study, accelerated venous maturation was observed in 26.5% of cases of intrauterine growth restriction in contrast to 8.5% in normal pregnancies (0.019).^39^ Accelerated villous maturation also occur more frequently in cases of fetal death beyond 20 week’s gestation (20%) when compared to normal pregnancy (0%)(p<0.001).^38^ Cigarette smoking also affects placental maturation as assessed by ultrasound since more mature-looking placentas are found in smoking mothers.^40^

To put the interpretation of accelerated villous maturation in perspective one should be aware of a recent study where the intraobserver reliability was assessed in the examination of 80 placentas. Agreement on accelerated villous maturation was poor, in contrast to the overall moderate to excellent agreement on acute chorioamnionitis and villitis of unknown origin.^41^

Macroscopic infarction correlated with higher UtA PI values at 20-24 weeks and higher UA PI at 34-38 weeks, and negatively with BWZS, suggesting that this too can be a surrogate marker for MVM, when microscopic examination of the placenta is not available. Microscopic infarction and increased syncytial knots correlated with higher UtA PI values at both visits but not with any of the other Doppler markers of placental insufficiency. Microscopic infarction correlated strongly with BWZS but increased syncytial knots had a weak association. Decidual arteriopathy correlated with higher UtA PI at 20-24 weeks and with higher UA PI at 34-38 weeks at both visits, but had a weak association with BWZS, as did distal villous hypoplasia. Our data also confirm that increased perivillous fibrin and IEVT are not good markers for MVM as they showed no or weak correlations with any of the Doppler findings and showed weak effects on BWZS.

The composite diagnosis of MVM correlated strongly with BWZS and with high UtA and UA PI values. Hypoxia due to placental dysfunction is a common mechanism of death in stillbirths of structurally normal fetuses, as seen in a previous study where hypoxic myocardial damage was the attributable cause of death in 70% of cases.^42^ A hypoxic placental environment, resulting from absent or incomplete remodeling of the maternal spiral arteries in early pregnancy,^37^ leads to altered uterine and intervillous blood flow,^8^ and eventually to MVM. This explains the association between MVM and abnormal Doppler findings,^39,43^ as confirmed in this study. The differences in UtA and UA PI (and ORs for increased values) according to individual placental features were always larger than for the composite definition of MVM, suggesting that specific placental abnormalities (i.e., a small-for-gestation placenta, accelerated maturation and macro- or microscopic infarctions,) more strongly reflect elevated vascular resistance and fetoplacental affectation than a combination of any three of the seven possible MVM features. Allocating equal weight to features with weaker associations with Doppler findings and fetal growth for the definition of MVM (e.g., decidual arteriopathy, increased syncytial knots and distal villous hypoplasia) weakens the correlation between MVM and clinical signs of functional placental changes.

The poor correlation of RPH with UtA PI and UA PI suggests that, in our population, placental abruption is more likely an acute event, in contrast to RPH associated with medical diseases.^44–46^ Our finding supports a previous report that placental abruption was not associated with fetal growth restriction.^44–46^

The association of villous-stromal karyorrhexis with lower BWZS has been described and the association with higher UA PI values is understandable, as it reflects obliteration of fetal vessels within the placental villi. The association with higher UtA PI values in early gestation is however intriguing, as the cause is usually ascribed to pathology within the fetal circulation, rather than abnormal maternal circulatory adaptation.^47^ Villitis (mostly VUE) was the only inflammatory placental feature that correlated with UtA PI, but it did not correlate with BWZS. The lack of association with UA PI is in line with the literature.^48^

## Strength and limitations

To our knowledge, this is the first large prospective study describing individual macroscopic and microscopic features of placental examination in pregnancies with ultrasound dating before 20 weeks randomly selected from a geographically representative cohort of participants in a prospective study, with no selection based on obstetric risk profile. This is also the first large prospective study comparing individual macroscopic and microscopic features of placental examination with BWZS and Doppler studies of the UtA, UA and MCA in mid and late pregnancy.

The data were collected some time ago, but the analysis is still valid as basic information was collected prospectively in a community that has not changed much in recent years. Although the study was designed to be large enough to address the effects of alcohol use during pregnancy on stillbirths and sudden infant death syndrome, the information collected on this randomly selected subset is sufficient and unbiased to assess more common outcomes like placental pathology, and their relation to Doppler findings and fetal growth.

A limitation is that our findings were not related to underlying medical diseases or other pregnancy outcomes since our intention was to focus on the basic findings. We also did not address missing data as the study contains large numbers in most of the categories. Recruiting only the first five interested women per day from all who were waiting for antenatal care could have introduced selection bias as women who present early in the morning for their antenatal visit may differ from those arriving later in the day. Also, as the GA was determined by ultrasound biometry up to 20 weeks, this may have underestimated the true GA and overestimated the BWZS for fetuses who were already growth restricted at the time of the first ultrasound examination.^30^ Another limitation is that the pathologists were aware of whether the pregnancy ended in a live birth or stillbirth and that the cohort with Doppler assessment at 34-38 weeks excluded all severely preterm births (indicated or spontaneous) and most of the stillbirths, thereby representing pregnancies at lower risk of pathology and weakening the correlations.

## Conclusions

Currently accepted features of MVM vary considerably in their associations with higher vascular resistance in the UtA and UA and with lower BWZS. The strongest features are a small-for-GA placenta, accelerated maturation and macroscopic or microscopic infarction. We suggest that these four findings should carry more weight in diagnosing MVM rather than being allocated the same weight as other features in the current MVM classification.

As placental weight and infarctions can be assessed macroscopically and both correlate well with BWZS and clinical markers of MVM (increased uteroplacental resistance), macroscopic assessment of the placenta is encouraged in settings where histologic examination is unavailable, as it helps in identifying pregnancies complicated by MVM, thereby creating the opportunity to guide management in future pregnancies.

## Figures and Tables

**Table 1.** Comparison of the background characteristics of the three study groups

	Placenta and Doppler PI at 20-24 weeks *N*=1022)		Placenta and Doppler PI at 34-38 weeks (*N*=979)		Placenta and birthweight z-score (*N*=1035)	
Variable	*N*	Mean (SD)	*N*	Mean (SD)	*N*	Mean (SD)
Maternal age (years)	1022	24.5 (5.8)	979	24.4 (5.8)	1035	24.7 (5.8)
Maternal weight (kg)	1009	63.1 (15.3)	967	63.7 (15.8)	1021	63.9 (15.7)
Mid-upper-arm circumference (mm)	1001	275 (47)	959	276 (47)	1014	278 (48)
Body Mass Index (kg/m^2^)	996	25.0 (5.8)	955	25.2 (6.0)	1007	25.4 (6.0)
Education (years)	1021	10.1 (1.7)	977	10.2(1.7)	1034	10.1(1.7)
Income (ZAR/month)	761	908 (612)	733	922 (607)	777	931 (615)
Birthweight (g)	894	3000 (596)	830	3102 (46)	1035	3017 (587)
Gestational age at birth (days)	894	272 (17)	830	275(10)	1035	272 (16)
Birthweight z-scores	894	−0.34 (1.05)	830	−0.29 (1.00)	1035	−0.31 (1.00)
Interval test-delivery (days)	894	113.9 (17.5)	830	29.8 (11.5)	NA	NA
Variable	*N*	%	*N*	%	*N*	%
Born < 34 weeks	39	3.8	0	0.0	37	3.6
Stillbirths	14	1.4	3	0.3	11	1.1

SD = standard deviation, ZAR = South African Rand (1 ZAR = 0.042 pound sterling or 0.053 United States dollar); NA = not applicable.

**Table 2.** Comparison of the prevalence of abnormal placental findings in the different cohorts

	Placenta and Doppler PI at 20-24 weeks (*N*=1022)	Placenta and Doppler PI at 34-38 weeks (*N*=979)	Birthweight z-score (*N*=1035)
Variable	*N* (%)	*N* (%)	*N* (%)
**Small placenta for gestational age** ¶	531 (53.1%)	500 (52.3%)	518 (51.1%)
**Macroscopic pathological infarction**	28 (2.8%)	22 (2.3%)	27 (2.6%)
**Decidual arteriopathy**	86 (8.4%)	79 (8.1%)	86 (8.3%)
**Accelerated maturation**	56 (5.5%)	32 (3.3%)	53 (5.1%)
**Increased syncytial knots**	523 (51.2%)	525 (53.6%)	531 (51.3%)
**Distal villous hypoplasia**	93 (9.1%)	89 (9.1%)	96 (9.3%)
**Increased perivillous fibrin** *	504 (49.3%)	497 (50.8%)	511 (49.4%)
**Increased extravillous trophoblast/fibrinoid islands** *	298 (29.2%)	282 (28.8%)	295 (28.5%)
**Microscopic pathological infarction**	88 (8.6%)	79 (8.1%)	90 (8.7%)
**Maternal vascular malperfusion** ‡	402 (39.6%)	384 (39.6%)	400 (39.0%)
**Macroscopic retroplacental hemorrhage**	73 (7.1%)	69 (7.1%)	74 (7.2%)
**Microscopic retroplacental hemorrhage**	102 (10.0%)	98 (10.0%)	102 (9.9%)
**Any retroplacental hemorrhage**	135 (13.2%)	134 (13.7%)	140 (13.5%)
**Thrombosis of cord vessels**	12 (1.2%)	15 (1.5%)	14 (1.4%)
**Avascular villi**	218 (21.3%)	213 (21.8%)	227 (21.9%)
**Chorionic plate/stem villous thrombosis**	103 (10.1%)	99 (10.1%)	102 (9.9%)
**Villous-stromal karyorrhexis**	273 (26.7%)	270 (27.6%)	275 (26.6%)
**Fetal vascular malperfusion** §	147 (14.4%)	144 (14.7%)	150 (14.5%)
**Chorioamnionitis**	325 (31.8%)	298 (30.4%)	311 (30.1%)
**Villitis**	245 (24.0%)	216 (22.1%)	241 (23.3%)

‡ 3 or more features of maternal vascular malperfusion;

§ 2 or more features of fetal vascular malperfusion;

¶ trimmed placental weight < 10^th^ percentile;

* no longer regarded as part of maternal vascular malperfusion.

**Table 3.** Association between placental pathology and uterine artery pulsatility indices

		At 20-24 weeks’ gestation			At 34-38 weeks’ gestation		
		*N*	Mean (SD)	OR (95% CI)	*N*	Mean (SD)	OR (95 %CI)
**Macroscopic features of maternal vascular malperfusion**							

Small placenta for gestational age¶	Yes	499	1.01 (0.35)	**4.86** **(2.94, 8.04)**	445	0.77 (0.21)	**5.92** **(2.81, 12.47)**
	
	No	431	0.87 (0.26)		411	0.70 (0.18)	

Macroscopic pathological infarction	Yes	25	1.28 (0.58)	**6.08** **(2.88, 12.82)**	19	0.77 (0.24)	2.52 (0.33, 19.44)
	
	No	920	0.94 (0.30)		853	0.73 (0.20)	

**Microscopic features of maternal vascular malperfusion**							

Decidual arteriopathy	Yes	83	1.04 (0.40)	**2.23** **(1.24, 3.99)**	71	0.77 (0.22)	2.5 (0.82, 7.67)
	
	No	864	0.94 (0.31)		803	0.73 (0.19)	

Accelerated maturation	Yes	51	1.34 (0.59)	**11.68** **(5.84, 23.37)**	30	0.89 (0.32)	**18.46** **(4.84, 70.38)**
	
	No	896	0.92 (0.28)		844	0.73 (0.19)	

Increased syncytial knots	Yes	483	0.97 (0.34)	**1.75** **(1.15, 2.67)**	463	0.76 (0.21)	**3.28** **(1.61, 6.67)**
	
	No	464	0.92 (0.29)		411	0.71 (0.17)	

Distal villous hypoplasia	Yes	84	1.01 (0.37)	**1.82** **(1, 3.33)**	78	0.78 (0.25)	**3.12** **(1.08, 8.95)**
	
	No	863	0.94 (0.31)		796	0.73 (0.19)	

Increased perivillous fibrin*	Yes	474	0.96 (0.33)	**1.43** **(0.95, 2.16)**	441	0.74 (0.21)	1.26 (0.64, 2.49)
	
	No	473	0.93 (0.30)		433	0.73 (0.18)	

Increased extravillous trophoblast/fibrinoid islands*	Yes	276	0.98 (0.35)	**1.63** **(1.07, 2.5)**	247	0.75 (0.22)	1.96 (0.95, 4.08)
	
	No	671	0.93 (0.30)		627	0.73 (0.18)	

Microscopic pathological infarction	Yes	80	1.20 (0.52)	**6.84** **(3.79, 12.34)**	70	0.84 (0.26)	**10.9** **(3.93, 30.23)**
	
	No	867	0.92 (0.28)		804	0.73 (0.19)	

Maternal vascular malperfusion‡	Yes	372	1.01 (0.38)	**3.23** **(2.07, 5.06)**	336	0.76 (0.21)	**2.67** **(1.33, 5.34)**
	
	No	568	0.90 (0.26)		531	0.72 (0.18)	

**Retroplacental hemorrhage**							

Macroscopic retroplacental hemorrhage	Yes	67	1.00 (0.39)	**1. 7** **(0.87, 3.33)**	60	0.74 (0.23)	1.23 (0.33, 4.55)
	
	No	880	0.94 (0.31)		814	0.73 (0.19)	

Microscopic retroplacental hemorrhage	Yes	94	0.91 (0.31)	**0.64** **(0.3, 1.36)**	89	0.71 (0.20)	0.41 (0.12, 1.42)
	
	No	853	0.95 (0.32)		785	0.74 (0.20)	

Any retroplacental hemorrhage	Yes	125	0.95 (0.34)	**1.0** **(0.55, 1.81)**	120	0.72 (0.20)	0.6 (0.21, 1.7)
	
	No	822	0.95 (0.31)		754	0.74 (0.19)	

**Fetal vascular malperfusion**							

Thrombosis of cord	Yes	11	0.90 (0.34)	**0.58** **(0.07, 5.16)**	13	0.66 (0.15)	0.07 (0, 2.7)
	
	No	934	0.95 (0.32)		859	0.74 (0.20)	

Avascular villi	Yes	203	0.98 (0.32)	**1.46** **(0.92, 2.31)**	192	0.74 (0.21)	1.35 (0.61, 3.01)
	
	No	744	0.94 (0.32)		682	0.73 (0.19)	

Chorionic plate/stem villous thrombosis	Yes	100	0.92 (0.30)	**0.73** **(0.36, 1.49)**	94	0.71 (0.17)	0.42 (0.13, 1.4)
	
	No	847	0.95 (0.32)		780	0.74 (0.20)	

Villous, stromal karyorrhexis	Yes	250	1.00 (0.37)	**1.88** **(1.22, 2.91)**	243	0.75 (0.22)	2.02 (0.97, 4.21)
	
	No	697	0.93 (0.29)		631	0.73 (0.18)	

Fetal vascular malperfusion§	Yes	134	0.99 (0.33)	**1.52** **(0.9, 2.57)**	132	0.72 (0.20)	0.69 (0.26, 1.84)
	
	No	812	0.94 (0.31)		741	0.74 (0.19)	

**Features of inflammation**							

Chorioamnionitis	Yes	311	0.94 (0.35)	**0.94** **(0.61, 1.45)**	271	0.72 (0.18)	0.54 (0.25, 1.15)
	
	No	636	0.95 (0.30)		603	0.74 (0.20)	

Villitis	Yes	228	1.00 (0.36)	**1.85** **(1.19, 2.87)**	196	0.77 (0.22)	**3.3** **(1.53, 7.11)**
	
	No	719	0.93 (0.30)		678	0.72 (0.19)	

SD standard deviation; OR = odds ratio, with bold indicating significance;

‡ 3 or more features of maternal vascular malperfusion excluding IPF and IEVT;

§ 2 or more features of fetal vascular malperfusion;

¶ trimmed placental weight < 10th percentile;

* no longer regarded as part of maternal vascular malperfusion.

**Table 4.** Association between placental pathology and umbilical artery pulsatility indices

		At 20-24 weeks’ gestation			At 34-38 weeks’ gestation		
		*N*	Mean (SD)	OR (95% CI)	*N*	Mean (SD)	OR (95% CI)
**Macroscopic features of maternal vascular malperfusion**							

Small placenta for gestational age¶	Yes	498	1.24 (0.18)	**5.33** **(2.46, 11.52)**	443	0.93 (0.16)	**27.01** **(10.2, 71.56)**
	
	No	456	1.19 (0.16)		397	0.86 (0.14)	

Macroscopic pathological infarction	Yes	26	1.23 (0.22)	**1.82** **(0.2, 16.15)**	17	0.97 (0.20)	**17.02** **(1.03, 281.6)**
	
	No	946	1.21 (0.17)		841	0.89 (0.15)	

**Microscopic features of maternal vascular malperfusion**							

Decidual arteriopathy	Yes	81	1.23 (0.19)	**1.9** **(0.53, 6.84)**	74	0.93 (0.17)	**5.97** **(1.36, 26.15)**
	
	No	893	1.21 (0.17)		786	0.89 (0.15)	

Accelerated maturation	Yes	49	1.26 (0.27)	**4.12** **(0.88, 19.32)**	26	0.94 (0.20)	5.95 (0.55, 64.2)
	
	No	925	1.21 (0.17)		834	0.89 (0.15)	

Increased syncytial knots	Yes	491	1.22 (0.18)	**2.04** **(0.98, 4.24)**	456	0.90 (0.15)	1.39 (0.58, 3.33)
	
	No	483	1.20 (0.16)		404	0.89 (0.15)	

Distal villous hypoplasia	Yes	87	1.24 (0.16)	**2.47** **(0.72, 8.44)**	74	0.93 (0.16)	**4.97** **(1.13, 21.9)**
	
	No	887	1.21 (0.17)		786	0.89 (0.15)	

Increased perivillous fibrin*	Yes	477	1.22 (0.18)	**1.66** **(0.8, 3.44)**	432	0.89 (0.16)	0.99 (0.42, 2.36)
	
	No	497	1.21 (0.17)		428	0.89 (0.15)	

Increased extravillous trophoblast/ fibrinoid islands*	Yes	280	1.23 (0.17)	**2.17** **(0.98, 4.82)**	243	0.89 (0.16)	0.98 (0.37, 2.58)
	
	No	694	1.21 (0.17)		617	0.89 (0.15)	

Microscopic pathological infarction	Yes	81	1.23 (0.19)	**1.78** **(0.49, 6.44)**	64	0.91 (0.18)	2.19 (0.44, 11.02)
	
	No	893	1.21 (0.17)		796	0.89 (0.15)	

Maternal vascular malperfusion‡	Yes	371	1.23 (0.19)	**2.53** **(1.2, 5.41)**	328	0.91 (0.16)	**2.62** **(1.07, 6.44)**
	
	No	596	1.20 (0.16)		524	0.88 (0.15)	

**Retroplacental haemorrhage**							

Macroscopic retroplacental haemorrhage	Yes	68	1.20 (0.17)	**0.64** **(0.15, 2.72)**	58	0.87 (0.18)	0.27 (0.04, 1.67)
	
	No	906	1.21 (0.17)		802	0.89 (0.15)	

Microscopic retroplacental haemorrhage	Yes	99	1.19 (0.18)	**0.4** **(0.12, 1.38)**	84	0.88 (0.16)	0.5 (0.11, 2.21
	
	No	875	1.22 (0.17)		776	0.89 (0.15)	

Any retroplacental haemorrhage	Yes	128	1.19 (0.18)	**0.39** **(0.13, 1.17)**	115	0.88 (0.16)	0.52 (0.14, 1.92)
	
	No	846	1.22 (0.17)		745	0.89 (0.15)	

**Fetal vascular malperfusion**							

Thrombosis of cord	Yes	11	1.26 (0.11)	**3.8** **(0.17, 87.05)**	15	0.82 (0.12)	0.03 (0, 1.2)
	
	No	961	1.21 (0.17)		843	0.89 (0.15)	

Avascular villi	Yes	206	1.21 (0.17)	**0.95** **(0.39, 2.3)**	181	0.89 (0.15)	0.8 (0.28, 2.34)
	
	No	768	1.21 (0.17)		679	0.89 (0.16)	

Chorionic plate/stem villous thrombosis	Yes	100	1.19 (0.20)	**0.48** **(0.14, 1.65)**	96	0.87 (0.17)	0.27 (0.06, 1.14)
	
	No	874	1.22 (0.17)		764	0.90 (0.15)	

Villous, stromal karyorrhexis	Yes	262	1.23 (0.18)	**2.62** **(1.16, 5.91)**	235	0.89 (0.16)	1.04 (0.39, 2.75)
	
	No	712	1.21 (0.17)		625	0.89 (0.15)	

Fetal vascular malperfusion§	Yes	138	1.20 (0.17)	**0.55** **(0.19, 1.59)**	131	0.87 (0.16)	0.4 (0.12, 1.39)
	
	No	835	1.22 (0.17)		728	0.90 (0.15)	

**Features of inflammation**							

Chorioamnionitis	Yes	313	1.20 (0.17)	**0.57** **(0.26, 1.25)**	263	0.88 (0.15)	0.6 (0.23, 1.57)
	
	No	661	1.22 (0.17)		597	0.90 (0.16)	

Villitis	Yes	234	1.20 (0.17)	**0.59** **(0.25, 1.39)**	195	0.89 (0.16)	1.01 (0.36, 2.84)
	
	No	740	1.22 (0.17)		665	0.89 (0.15)	

SD = standard deviation; OR = odds ratio, with bold indicating significance;

‡ 3 or more features of maternal vascular malperfusion;

§ 2 or more features of fetal vascular malperfusion;

¶ trimmed placental weight < 10th percentile;

* no longer regarded as part of maternal vascular malperfusion.

**Table 5.** Association between placental pathology and middle cerebral artery pulsatility indices

		At 20-24 weeks’ gestation			At 34-38 weeks’ gestation		
		*N*	Mean (SD)	OR (95% CI)	*N*	Mean (SD)	OR (95% CI)
**Macroscopic features of maternal vascular malperfusion**							

Small placenta for gestational age¶	Yes	431	1.69 (0.27)	**0.89** **(0.52, 1.53)**	430	1.73 (0.31)	**0.54** **(0.35, 0.84)**
	
	No	384	1.69 (0.24)		401	1.79 (0.31)	

Macroscopic pathological infarction	Yes	22	1.75 (0.27)	**2.32** **(0.53, 10.07)**	18	1.79 (0.35	1.3 (0.29, 5.71)
	
	No	805	1.69 (0.25)		829	1.76 (0.31)	

**Microscopic features of maternal vascular malperfusion**							

Decidual arteriopathy	Yes	68	1.71 (0.27)	**1.33** **(0.52, 3.42)**	70	1.76 (0.31)	0.97 (0.44, 2.11)
	
	No	761	1.69 (0.25)		779	1.76 (0.31)	

Accelerated maturation	Yes	44	1.65 (0.30)	**0.53** **(0.14, 1.91)**	25	1.72 (0.40)	0.62 (0.17, 2.24)
	
	No	785	1.69 (0.25)		824	1.76 (0.31)	

Increased syncytial knots	Yes	400	1.70 (0.26)	**1.33** **(0.77, 2.27)**	448	1.78 (0.31)	1.46 (0.95, 2.25)
	
	No	429	1.68 (0.25)		401	1.74 (0.32)	

Distal villous hypoplasia	Yes	70	1.69 (0.28)	**1.07** **(0.41, 2.78)**	79	1.82 (0.34)	1.89 (0.9, 3.94)
	
	No	759	1.69 (0.25)		770	1.76 (0.31)	

Increased perivillous fibrin*	Yes	399	1.69 (0.24)	**0.93** **(0.54, 1.58)**	427	1.77 (0.31)	1.25 (0.81, 1.92)
	
	No	430	1.69 (0.27)		422	1.75 (0.31)	

Increased extravillous trophoblast/fibrinoid islands*	Yes	227	1.69 (0.25)	**1** **(0.55, 1.82)**	238	1.76 (0.32)	1.04 (0.64, 1.67)
	
	No	602	1.69 (0.26)		611	1.76 (0.31)	

Microscopic pathological infarction	Yes	68	1.69 (0.29)	**0.96** **(0.36, 2.56)**	67	1.70 (0.34)	0.48 (0.21, 1.09)
	
	No	761	1.69 (0.25)		782	1.77 (0.31)	

Maternal vascular malperfusion‡	Yes	310	1.69 (0.26)	**1.07** **(0.62, 1.87)**	324	1.75 (0.31)	0.87 (0.56, 1.35)
	
	No	513	1.69 (0.25)		518	1.77 (0.32)	

**Retroplacental hemorrhage**							

Macroscopic retroplacental hemorrhage	Yes	60	1.75 (0.30)	**2.6** **(1.04, 6.53)**	60	1.68 (0.32)	0.42 (0.18, 1.0)
	
	No	769	1.68 (0.25)		789	1.77 (0.31)	

Microscopic retroplacental hemorrhage	Yes	85	1.70 (0.28)	**1.14** **(0.48, 2.72)**	88	1.71 (0.30)	0.52 (0.26, 1.07)
	
	No	744	1.69 (0.25)		761	1.77 (0.31)	

Any retroplacental hemorrhage	Yes	113	1.72 (0.28)	**1.73** **(0.83, 3.63)**	118	1.71 (0.31)	**0.53** **(0.28, 0.99)**
	
	No	716	1.68 (0.25)		731	1.77 (0.31)	

**Features of fetal vascular malperfusion**							

Thrombosis of cord	Yes	11	1.76 (0.16)	**2.55** **(0.34, 19.03)**	12	1.81 (0.30)	1.54 (0.25, 9.42)
	
	No	816	1.69 (0.26)		835	1.76 (0.31)	

Avascular villi	Yes	175	1.69 (0.24)	**0.95** **(0.49, 1.84)**	186	1.78 (0.33)	1.24 (0.74, 2.07)
	
	No	654	1.69 (0.26)		663	1.76 (0.31)	

Chorionic plate/stem villous thrombosis	Yes	93	1.71 (0.26)	**1.32** **(0.58, 3)**	90	1.73 (0.26)	0.66 (0.33, 1.34)
	
	No	736	1.69 (0.25)		759	1.77 (0.32)	

Villous, stromal karyorrhexis	Yes	204	1.70 (0.27)	**1.14** **(0.62, 2.11)**	221	1.77 (0.32)	1.18 (0.72, 1.92)
	
	No	625	1.69 (0.25)		628	1.76 (0.31)	

Fetal vascular malperfusion§	Yes	118	1.69 (0.24)	**0.98** **(0.45, 2.1)**	122	1.78 (0.31)	1.2 (0.65, 2.2)
	
	No	710	1.69 (0.26)		726	1.76 (0.31)	

**Features of inflammation**							

Chorioamnionitis	Yes	267	1.69 (0.28)	**1.09** **(0.61, 1,92)**	259	1.74 (0.31)	0.72 (0.45, 1.15)
	
	No	562	1.69 (0.24)		590	1.77 (0.32)	

Villitis	Yes	201	1.68 (0.25)	**0.85** **(0.45, 1.6)**	190	1.76 (0.33)	0.95 (0.57, 1.59)
	
	No	628	1.69 (0.26)		659	1.76 (0.31)	

SD standard deviation; OR = odds ratio, with bold indicating significance;

‡ 3 or more features of maternal vascular malperfusion;

§ 2 or more features of fetal vascular malperfusion;

¶ trimmed placental weight < 10th percentile;

* no longer regarded as part of maternal vascular malperfusion.

**Table 6.** Association between placental pathology and birthweight z-scores

		*N*	Mean BWZS ±SD	OR (95% CI)
**Macroscopic features of maternal vascular malperfusion**				
Small placenta for gestational age¶	Yes	518	−0.77 (0.97)	**0.31** **(0.26, 0.37)**
	No	495	0.18 (0.89)	
Macroscopic pathological infarction	Yes	27	−0.87 (1.64)	**0.62** **(0.45, 0.86)**
	No	1006	−0.29 (1.02)	
**Microscopic features of maternal vascular malperfusion**				
Decidual arteriopathy	Yes	86	−0.53 (1.21)	**0.8** **(0.65, 0.98)**
	No	949	−0.29 (1.02)	
Accelerated maturation	Yes	53	−0.86 (1.46)	**0.61** **(0.47, 0.78)**
	No	982	−0.28 (1.01)	
Increased syncytial knots	Yes	531	−0.41 (1.07)	**0.81** **(0.72, 0.92)**
	No	504	−0.19 (1.00)	
Distal villous hypoplasia	Yes	96	−0.63 (1.05)	**0.73** **(0.6, 0.89)**
	No	939	−0.27 (1.04)	
Increased perivillous fibrin*	Yes	511	−0.42 (1.06)	**0.82** **(0.72, 0.92)**
	No	524	−0.20 (1.01)	
Increased extravillous trophoblast/fibrinoid islands*	Yes	295	−0.55 (1.11)	**0.73** **(0.64, 0.83)**
	No	740	−0.21 (1.00)	
Microscopic pathological infarction	Yes	90	−0.80 (1.23)	**0.62** **(0.51, 0.76)**
	No	945	−0.26 (1.01)	
Maternal vascular malperfusion‡	Yes	400	−0.64 (1.07)	**0.57** **(0.5, 0.66)**
	No	627	−0.09 (0.96)	
**Retroplacental hemorrhage**				
Macroscopic retroplacental hemorrhage	Yes	74	−0.20 (1.24)	1.12 (0.89, 1.4)
	No	961	−0.31 (1.02)	
Microscopic retroplacental hemorrhage	Yes	102	−0.27 (1.09)	1.04 (0.85, 1.36)
	No	933	−0.31 (1.04)	
Any retroplacental hemorrhage	Yes	140	−0.25 (1.11)	1.06 (0.89, 1.26)
	No	895	−0.32 (1.03)	
**Features of fetal vascular malperfusion**				
Thrombosis of cord	Yes	14	0.14 (0.75)	1.53 (0.91, 2.56)
	No	1020	−0.31 (1.04}	
Avascular villi	Yes	227	−0.33 (1.05)	0.98 (0.85, 1.12)
	No	808	−0.30 (1.04)	
Chorionic plate/stem villous thrombosis	Yes	102	−0.15 (1.16)	1.18 (0.97, 1.44)
	No	933	−0.32 (1.03)	
Villous-stromal karyorrhexis	Yes	275	−0.44 (1.14)	**0.85** **(0.74, 0.97)**
	No	760	−0.26 (1.00)	
Fetal vascular malperfusion§	Yes	150	−0.32 (1.13)	0.98 (0.83, 1.16)
	No	885	−0.30 (1.03)	
**Features of inflammation**				
Chorioamnionitis	Yes	311	−0.30 (1.06)	1.01 (0.89, 1.15)
	No	724	−0.31 (1.04)	
Villitis	Yes	241	−0.39 (1.15)	0.9 (0.79, 1.03)
	No	794	−0.28 (1.00)	

BWZS = birthweight z-score; SD = standard deviation; OR = odds ratio, with bold indicating significance;

‡ 3 or more features of maternal vascular malperfusion;

§ 2 or more features of fetal vascular malperfusion;

¶ trimmed placental weight < 10th percentile;

* no longer regarded as part of maternal vascular malperfusion.
